# Empowering the Patient Voice and Optimizing HIV Care in the Middle East

**DOI:** 10.7759/cureus.96078

**Published:** 2025-11-04

**Authors:** Sawsan AlMadhi, Ahmad Alharbi, Bertho Makso, Jameela Al Salman, Jehad Abdalla, Mehdi Karkouri

**Affiliations:** 1 Advocacy, AlignnEficient Health Consultancies, Dubai, ARE; 2 Medicine, King Abdulaziz Medical City, Riyadh, SAU; 3 Regional Community Engagement &amp; Networks, Proud Lebanon, Beirut, LBN; 4 Infectious Disease, King Hamad American Mission Hospital, A'ali, BHR; 5 Infectious Disease, Sheikh Shakhbout Medical City, Abu Dhabi, ARE; 6 Pathology, Ibn Rochd University Hospital Center, Casablanca, MAR; 7 Pathology, Hassan II University of Casablanca, Casablanca, MAR

**Keywords:** hiv policy, hiv stigma, human immunodeficiency virus, patient advocacy groups, patient voice

## Abstract

The Middle East is in a critical phase of its regional human immunodeficiency virus (HIV) response. Infections continue to rise at an alarming rate, and there are significant challenges that hinder the delivery of effective HIV care, such as stigma, lack of awareness, and inadequate access to prevention, testing, and management services. Stigma and discrimination against people living with HIV are pervasive and exacerbated by misinformation. This often impedes linkage to care, thereby promoting the spread of the virus and leading to worsened public health outcomes. There is an urgent need to elevate the patient voice in the context of HIV care in the region, to humanize the epidemic and combat stigma and misinformation. Additionally, the role of patient advocacy groups in the region should be expanded, and various stakeholders, including policymakers and healthcare authorities, should be engaged to optimize the HIV care pathway. We present 15 recommendations aimed at different stakeholders to help achieve these crucial objectives.

## Editorial

Introduction

The Middle East and North Africa (MENA) is one of only two regions in the world where human immunodeficiency virus (HIV) cases are still increasing. Between 2010 and 2022, there was a 61% increase in new HIV cases, with 17,000 new infections recorded in 2022 alone [1]. Furthermore, only 38% of people living with HIV (PLWH) accessed antiretroviral therapy (ART) in the region in 2019 [2]. The Joint United Nations Programme on HIV/AIDS primarily attributes this to inadequate prevention services for marginalized populations, which is exacerbated by exclusionary regulations and social discrimination against PLWH [3]. These trends starkly contrast with the global decline in new HIV infections, underscoring the unique challenges faced within the region.

Several factors account for the unique challenges in HIV management. Key populations, including men who have sex with men, people who inject drugs, and sex workers, are disproportionately affected [4,5]. These groups face significant social stigma and legal challenges, which hinder their access to prevention, testing, and treatment services. In many countries in the region, behaviors associated with these populations are criminalized, exacerbating stigma and discrimination against PLWH. Furthermore, there is a general paucity of region-specific HIV data due to underfunding, inadequate surveillance systems, and complications caused by geopolitical and humanitarian crises [1,4].

The regional sociocultural nuances significantly hamper efforts to address the HIV epidemic effectively. Stigma and discrimination against PLWH are pervasive and influenced by cultural, religious, and social norms. In many societies, HIV is associated with behaviors considered taboo, leading to discrimination and social isolation. Within this regional sociocultural context, HIV is deprioritized in many countries due to competition with other urgent priorities [5]. Moreover, the legal environment in many Middle Eastern countries criminalizes behaviors such as drug use, same-sex relations, and sex work. These regulations prevent at-risk populations from accessing HIV prevention and treatment services [1]. The criminalization of these behaviors also makes it difficult to gather accurate epidemiological data, further complicating efforts to control the spread of HIV within the region [6]. A more tolerant legal environment backed by political commitment is required to enhance the regional HIV response and address the unmet needs of at-risk populations [7].

The aim of this manuscript is to present an overview of HIV management in the Middle East, with a special focus on empowering patient voices, combating stigma, and enhancing the roles of patient advocacy groups. We also present recommendations to various stakeholders to optimize the management of HIV in the region.

Challenges in the HIV care continuum

Stigma and discrimination are pervasive in the Middle East region, severely impacting all stages of the HIV care continuum. PLWH face social ostracism, discrimination in healthcare settings, and legal repercussions, such as deportation for expatriates, due to their HIV status. These factors discourage individuals from seeking testing, care, and treatment, perpetuating the cycle of infection and poor health outcomes. Logistical barriers in some countries, such as limited healthcare infrastructure in some regions and transportation issues, further complicate HIV care. In many areas, healthcare facilities are not easily accessible, and there are few specialized HIV care providers, particularly in rural regions. HIV programs in the region are also severely underfunded. Limited financial resources in some countries hinder the implementation of comprehensive prevention, testing, and treatment initiatives [4]. Additionally, inadequate surveillance systems fail to capture accurate epidemiological data, making it difficult to allocate resources effectively [5]. Furthermore, public awareness and education about HIV remain insufficient. Misconceptions about HIV transmission, prevention, and treatment persist, contributing to stigma and discrimination. Comprehensive sexual education programs are often lacking, and public health campaigns are currently not sufficiently robust to address the widespread misinformation [1,4,5]. However, it is noteworthy that there are marked differences in the standards of care among countries in the region. Some countries have sophisticated care centers that are capable of delivering high-quality HIV care, whereas other regions lag behind in their national HIV response.

Aim

To address the various challenges in the regional HIV response, we propose four key objectives. These objectives align with the overarching aims of optimizing HIV management, improving health outcomes, and combating stigma against PLWH in the Middle East.

Objective 1: Empowering Patient Voices

The term “patient voice” in healthcare refers to the active inclusion of patient perspectives, preferences, experiences, and insights into their healthcare management and the broader healthcare system. It signifies a shift from a traditional model of healthcare, where decisions were predominantly made by healthcare providers, to a more collaborative and patient-centered approach. This concept emphasizes the importance of relying on patient input and integrating feedback into care planning and decision-making processes to improve health outcomes and patient satisfaction [8].

The patient voice is particularly significant in the context of HIV care. Empowering the voices of PLWH is crucial as it helps to humanize the epidemic, reducing stigma and fostering understanding. It also ensures that healthcare interventions are tailored to the needs and preferences of PLWH, which can enhance treatment adherence and overall quality of care. Research has demonstrated that when patients are actively involved in their care, there are improvements in health outcomes, including better adherence to ART and increased retention in care [9,10]. In addition to including PLWH in their treatment plans, the patient voice is a powerful tool that can be leveraged in policymaking and advocacy, which may enhance the overall quality of HIV care in the region.

Objective 2: Combating Stigma and Discrimination

Stigma and discrimination against PLWH are pervasive in the Middle East region, deeply rooted in sociocultural nuances and conservative attitudes [4]. This profoundly impacts the mental health, quality of life, and self-worth of PLWH, creating barriers to accessing care and adhering to treatment [6]. The fear of being ostracized can deter individuals from seeking timely diagnosis and treatment, exacerbating the spread of the virus and leading to worsened health outcomes [11].

To combat stigma against PLWH in the region, we propose various strategies/initiatives targeted toward different groups. These strategies aim to address stigma across different levels, from internalized self-stigma to systemic discrimination against PLWH. Tailored toward healthcare providers, we suggest implementing mandatory HIV-related continuing medical education. We also recommend that public awareness campaigns be conducted via social media, print media, television, and radio to combat misinformation and stigma, and to humanize HIV by featuring the testimonials of PLWH from the region. Additionally, we recommend establishing antidiscriminatory legislation to protect the rights of PLWH and ensure fair access to healthcare, housing, and employment. Aimed toward PLWH, we recommend promoting counseling for comprehensive mental health support, promoting adherence, and addressing self-stigma. Finally, we also recommend the implementation of community-based programs such as volunteer support groups to minimize stigma among the general public.

Objective 3: Enhancing the Role of Patient Advocacy Groups

Patient advocacy groups play a crucial role in representing patients and elevating their voices, particularly in the context of chronic diseases like HIV. However, in the Middle East, the presence and effectiveness of patient advocacy groups are significantly hampered by several factors. There is a marked lack of funding and resources allocated to these organizations, which limits their capacity to operate and expand their reach. Furthermore, HIV-related issues are often deprioritized in the regional health agendas and overshadowed by other public health concerns. Systemic discriminatory attitudes toward HIV and those affected by it further exacerbate these challenges, creating an environment where patient advocacy groups struggle to gain the necessary support and recognition. This lack of robust patient advocacy infrastructure impedes the overall HIV response in the region, underscoring the urgent need for increased support and integration of patient advocacy groups.

Despite these challenges, some organizations such as Proud Lebanon and Manaah in Saudi Arabia operate as patient advocacy groups, conducting initiatives to elevate the voices of PLWH and protect their rights, and filling in gaps in healthcare systems. In the Middle East region, establishing and supporting patient advocacy groups is a crucial priority to enhance the HIV response. Patient advocacy groups can play a vital role in bridging gaps within healthcare systems by offering support services to PLWH and campaigning for their rights. However, the widespread systemic stigma and discriminatory attitudes toward HIV significantly hinder the establishment and effective functioning of such organizations. Allocating adequate funding and resources is essential to empowering such organizations to conduct activities effectively. Furthermore, there is a need for enhanced collaboration among patient advocacy groups, healthcare providers, and policymakers.

Research reinforces the transformative potential of HIV advocacy programs in changing social norms and improving health outcomes. A landmark systematic review comprising 25 studies demonstrated that HIV advocacy programs can effectively shift social norms by facilitating impactful changes in general awareness, attitudes, and public behavior. Advocacy programs were also shown to improve rates of voluntary counseling, HIV testing, and ART adherence. Key strategies included involving at-risk populations, working with peer advocates, and leveraging support from public figures [12]. Another systematic review of 20 studies from 1995 to 2014 concluded that support groups for PLWH significantly improve clinical outcomes, leading to increased retention in care, reduced morbidity, and enhanced quality of life [13]. These findings highlight the critical role of patient advocacy groups in supporting PLWH and in shaping public perceptions and policies. By providing comprehensive support and engaging in advocacy, patient advocacy groups can address both the practical and social challenges faced by PLWH, ultimately contributing to a more effective and compassionate HIV response in the region.

Objective 4: Optimizing the HIV Patient Care Pathway

Supporting robust prevention, testing, linkage to care, management, and follow-up strategies is crucial for controlling the spread of HIV in the region. To enhance HIV care delivery, a comprehensive and multifaceted approach is required from various stakeholders, including healthcare providers, patient advocacy groups, policymakers, and healthcare authorities. Key areas of focus should be expanding access to pre-exposure prophylaxis (PrEP), implementing self-testing programs, and establishing confidential testing clinics [14,15]. Additionally, strengthening peer support systems and patient navigation services can significantly improve linkage to care and treatment adherence. Integrating multidisciplinary care teams to provide holistic support for PLWH, alongside robust stigma reduction campaigns, is essential for long-term success. We hereby present recommendations jointly formulated by infectious disease specialists, policymakers, patient advocacy group representatives, and PLWH to optimize the HIV patient care pathway in the region.

Recommendations

To achieve the four key objectives presented, we provide a total of 15 specific recommendations encompassing prevention, testing, linkage to care, and management to optimize the regional HIV response. These recommendations are presented in Tables 1-3.

**Table 1:** Recommendations to optimize prevention and testing. HIV: human immunodeficiency virus; PrEP: pre-exposure prophylaxis.

Number	Recommendation	Description
1	Expand access to PrEP	Increase the availability and accessibility of PrEP to at-risk populations through confidential HIV clinics.
2	Conduct public awareness campaigns	Leverage social media and engage reputed influencers to improve knowledge of HIV and its transmission and reduce HIV-associated stigma.
3	Implement self-testing programs	Develop and distribute HIV self-testing kits to ensure privacy and convenience, thereby encouraging individuals to know their status.
4	Establish confidential testing clinics	Set up dedicated clinics offering confidential testing and additional services like PrEP counseling.
5	Conduct outreach testing and mobile testing	Deploy mobile testing units to reach at-risk populations in rural and underserved areas.

**Table 2:** Recommendations to optimize linkage to care and management. CME: continuing medical education; HIV: human immunodeficiency virus; PLWH: people living with HIV.

Number	Recommendation	Description
6	Provide integrated, multidisciplinary HIV care	Implement multidisciplinary care teams in clinical settings to provide integrated care, including medical treatment, mental health support, and social services.
7	Provide tailored education to healthcare providers	Deliver mandatory education for healthcare providers via online platforms as part of CME programs to improve knowledge and awareness and combat stigma against PLWH.
8	Enhance peer support and patient navigation	Train and employ peer navigators to assist PLWH in accessing and navigating healthcare services, improving linkage to care and treatment adherence.
9	Establish and fund patient advocacy groups	Allocate funding and resources to establish and strengthen patient advocacy groups, ensuring they can function effectively.
10	Establish dedicated support hotlines	Establish hotlines to provide information and referrals to at-risk individuals, linking them to necessary services.
11	Enhance the care pathway with digital tools	Utilize digital tools such as telemedicine for remote consultations and automated notifications to promote adherence and monitoring, and establish online support groups and communication channels to enhance patient support.

**Table 3:** Policy and research-related recommendations. HIV: human immunodeficiency virus; PLWH: people living with HIV.

Number	Recommendation	Description
12	Establish and enforce policies to protect PLWH	Implement policies to ensure fair access to healthcare, employment, marriage, and housing for PLWH, protecting them from discrimination and ensuring their rights are upheld.
13	Provide education to PLWH	Provide education to improve knowledge of their rights and to teach effective communication. This can be delivered via online or mobile platforms to maximize access.
14	Leverage electronic medical records to conduct region-specific HIV research	Conduct region-specific HIV research to understand the unique characteristics of the Middle East region in terms of epidemiology, transmission dynamics, drug resistance, and more.
15	Conduct regular conferences and scientific meetings	The purpose of these meetings is to serve as platforms for exchanging ideas, sharing updated data, and promoting best practices in the region.

Conclusion

Enhancing HIV care delivery in the Middle East requires concerted, multifaceted efforts to address the complex and diverse needs of PLWH. Elevating the voices of PLWH is crucial to ensuring that care is tailored to their unique experiences and challenges. The recommendations provided in this manuscript include expanding access to prevention tools like PrEP, implementing self-testing programs, and establishing confidential testing clinics. Strengthening peer support systems and patient navigation services, promoting integrated care models, and providing comprehensive mental health services are also recommended as important priorities. These recommendations, which were formulated based on the experiences of PLWH, focus on optimizing prevention, testing, linkage to care, and HIV management, and are aligned with the four overarching objectives: to empower patient voices, combat stigma and discrimination, enhance the role of patient advocacy groups, and optimize the patient care pathway.

Combating stigma and discrimination is especially important, and tailored efforts are needed in this regard. Public awareness campaigns, education for healthcare providers, and robust antidiscrimination policies are essential to creating a supportive environment for PLWH. By implementing these recommendations, we can improve health outcomes, reduce the incidence of new infections, and foster a more inclusive and supportive society. The collective effort of healthcare providers, policymakers, patient advocacy groups, and healthcare authorities is vital to achieving these goals and ensuring long-term success in the fight against HIV.
